# Fretting Wear Behaviors of Silicone Rubber under Dry Friction and Different Lubrication Conditions

**DOI:** 10.3390/ma17112598

**Published:** 2024-05-28

**Authors:** Ruotong Liu, Jie Su, Tengfei Zhang, Liaoliang Ke

**Affiliations:** School of Mechanical Engineering, Tianjin University, Tianjin 300350, China; lrt7868664@126.com (R.L.); tfzhang@tju.edu.cn (T.Z.)

**Keywords:** fretting wear, lubrication, silicone rubber, wear volume, wear mechanism

## Abstract

The fretting wear behaviors of silicone rubber under dry friction and different lubrication conditions are studied experimentally. Water, engine oil, dimethyl silicone oil (DSO), and dimethyl silicone oil doped with graphene oxide (DSO/GO) are selected as lubricants. Under the liquid lubrication conditions, the silicone rubber samples are always immersed in the same volume of lubricant. The contact model of a 440C steel ball and silicone rubber sample is the sphere-on-flat contact. The reciprocating fretting wear experiments are carried out using the reciprocating friction wear tester. A scanning electron microscope and three-dimensional white-light interference profilometer are used to detect the surface wear morphology and obtain the wear volume, respectively. The influences of normal force, lubrication condition, and displacement amplitude on fretting wear behavior are discussed. The fretting wear performances of silicone rubber under different fretting states and lubrication conditions are compared. The results show that for a small normal force, silicone rubber has the best wear resistance under DSO/GO lubrication. While for a large normal force, silicone rubber has the best wear resistance under engine oil lubrication.

## 1. Introduction

Silicone rubber is a common type of rubber whose molecular backbone is made up of silicon and oxygen atoms alternately [1]. It has many excellent mechanical properties, including high and low temperature resistance, high elasticity, wear resistance, aging resistance, good chemical stability, and corrosion resistance [2,3]. Thus, silicone rubber is widely used in sealing, bonding, isolation, and insulation fields [4,5,6,7]. Specifically, silicone rubber is one of the commonly used materials for dynamic seals. Reciprocating seals, as a type of dynamic seal, are extensively used in hydraulic systems, such as hydraulic actuators [8]. In order to prevent lubricant leakage, lubricating film with a certain thickness will be formed on the contact interface of the reciprocating seals. Therefore, many reciprocating seals always operate under liquid lubrication conditions. In addition, reciprocating seals are usually used in a vibration environment with small displacement amplitudes [9]. Therefore, fretting wear inevitably occurs on the contact interface of reciprocating seals [10,11]. It will cause damage and crack formation on the material surface. The wear of the surface will lead to changes in contact pressure, contact width, and lubricating film thickness, which are the main factors affecting the performance of seals. Therefore, wear on the contact interface can result in a decrease in sealing performance, specifically manifested as leakage or loosening of the seals [12,13,14]. Ultimately, it will cause the failure of the entire hydraulic system, leading to economic losses, resource waste, environmental pollution, and other issues [15,16]. As a result, it is essential to study the fretting wear behavior of silicone rubber under different lubrication conditions and minimize the fretting wear of a sealing contact surface as much as possible.

Fretting refers to the relative motion of contact surfaces with a very small displacement amplitude. Fretting wear refers to the damage and wear caused by fretting, and it creates micropits and causes a significant decrease in the quality of the contact surface, which increases the surface roughness. When two contact bodies are subjected to external compression and serve in a vibration environment, the fretting wear phenomenon usually occurs. There are many influence factors of fretting wear, mainly including material properties, contact forms, displacement amplitude, normal force, frequency, and external environment [17,18]. Fretting wear is a process involving multiple interrelated behaviors, including plastic deformation, crack formation and propagation, and debris evolution. It widely occurs in aerospace, mechanical processing, transportation, and other fields, such as interference fit assembly parts and electrical contact parts [19,20,21].

The friction and wear behaviors of hard materials such as metals and ceramics under liquid lubrication conditions have been extensively studied. Mi et al. [22] investigated the impact of temperature on fretting wear behaviors of 690 alloy in water. The results indicated that the wear volume of the material increases as water temperature increases. Under different lubrication conditions, Zhang et al. [23] analyzed the fretting wear behavior of 42CrMo4 and CuNiAl under a flat-on-flat contact model. The selected liquid lubricants were oil, artificial seawater, and filtered water. They found that the wear of the material was the least severe under oil lubrication. Under different lubrication conditions, He et al. [24] discussed the sliding friction and wear behaviors of GCr15 bearing steel. The results indicated that when the lean lubrication transforms dry friction, the coefficient of friction (COF) and wear rate of the material underwent a sudden change. Chen et al. [25] studied the tribological properties of Si_3_N_4_-hBN ceramic composites and found that bovine serum lubrication had better tribological properties compared to physiological saline lubrication. Jia et al. [26] compared the sliding wear performance of three polymers, polytetrafluoroethylene (PTFE), polyphenylene sulfide (PPS), and polyamide 66 (PA66), under dry friction and liquid paraffin lubrication conditions. Their experimental results showed that under the liquid paraffin lubrication condition, the COF of all three materials decreased, the wear of PA66 increased, while the wear of PPS and PTFE decreased.

It is a remarkable fact that the fretting wear behavior of rubber materials under lubrication conditions has not been studied yet. At present, under liquid lubrication conditions, there are a small number of investigations on the sliding friction and wear behavior of rubber materials. Liu et al. [27] discussed the impacts of several typical lubricants on the sliding wear performance of nitrile rubber. They observed that rubber had the best friction and wear performance when lubricated with tung oil. Mofidi and Prakash [28] studied the two-body abrasive wear of four rubber materials used as elastic seals under lubrication and dry friction conditions. The selected lubricants were synthetic ester, polyalphaolefin, and mineral oil. Their results confirmed that the abrasive wear was more severe under the mineral oil lubrication condition in most cases. Feng et al. [29] discussed the influence of surface roughness on lubrication state and wear behavior of nitrile rubber by using reciprocating sliding wear tests. It was shown that as the surface roughness increases, the friction pair was more likely to enter a mixed lubrication state.

Common liquid lubricants include engine oil, silicone oil, paraffin liquid, ionic liquid, etc. Engine oil plays a necessary role in reducing friction, increasing lubrication, and in the cooling effects of engines [30,31]. As an environmentally friendly lubricant, dimethyl silicone oil (DSO) has many advantages, such as high thermal stability and chemical inertness. It is often used as a lubricant in aerospace and electronics industry fields [32]. Graphene is a typical two-dimensional nanomaterial. Due to the advantages of high strength, easy shearing, and a smooth surface, graphene is often used as an additive in liquid lubricants to improve their tribological performance [33,34]. However, graphene is prone to caking and precipitation in the lubricant, and it even leads to the serious wear of friction pairs. Graphene oxide (GO) is a derivative of graphene, which not only retains the advantages of graphene but also has better dispersion ability in liquids due to its surface containing a large amount of reactive oxygen species [35].

The main purpose of this paper is to investigate the fretting wear behaviors of silicone rubber under dry friction and different lubrication conditions. Water, engine oil, DSO, and dimethyl silicone oil doped with graphene oxide (DSO/GO) are selected as lubricants in the fretting experiment. A reciprocating friction wear tester is employed to conduct fretting wear experiments between a 440C steel ball and silicone rubber coating perfectly bonded to a S45C steel sheet. A scanning electron microscope (SEM) and three-dimensional white-light interference profilometer are later used to detect the surface wear morphology and determine the wear volume, respectively. The effects of lubrication condition, normal force, and displacement amplitude on the friction curves, COF, damage characteristics, and wear volume are studied. The wear performances of silicone rubber are compared under different lubrication conditions and fretting states. The fretting wear mechanism of silicone rubber is discussed under different lubrication conditions.

Compared with the relevant literature in this field, the novelties of this paper are given as follows: (1) The fretting wear performances of silicone rubber are compared under different normal forces, displacement amplitudes, and lubrication conditions. (2) The fretting wear mechanism of silicone rubber is discussed under different lubrication conditions. The forms of fretting wear include abrasive wear, fatigue wear, and adhesive wear. (3) It is found that the DSO/GO lubrication has the best lubrication effect when the fretting process is in the gross slip region, while the engine oil lubrication has the best lubrication effect when the fretting process runs in the partial slip region.

## 2. Materials and Fretting Wear Experimental Method

### 2.1. Materials

Silicone rubber is selected as the sample for fretting wear experiments in this paper. Its material properties are as follows: density 1.25 g/cm^3^, shore A hardness 65 Ha, surface roughness 0.13 μm, and Young’s modulus 11 MPa. The fretting experiment conditions include consideration of dry friction and liquid lubrication. Four typical liquid lubricants, water, engine oil, DSO, and DSO/GO, are selected.

The purity of GO used in this experiment is greater than 95 wt%. The properties of GO are the following: number of layers 1–5, thickness 1.0–1.77 nm, layer diameter 10–50 μm, and specific surface area 360–450 m^2^/g. The preparation method of DSO/GO is as follows: Take an appropriate amount of DSO and add the well-weighed GO and the dispersant (sodium dodecyl sulfate). The mass fraction of GO in DSO/GO is 0.1%. The mass ratio of GO to dispersant is 10:1. The mixture is magnetically stirred for 30 min. And then the resulting solution is transferred to the ultrasonic processor for ultrasonic treatment for 30 min in order to realize the uniform dispersion of GO into DSO [36]. The engine oil is supplied by Petrochina Co., Ltd., Beijing, China; and DSO is supplied by Dow Corning company, Midland, MI, USA. GO is provided by Suzhou Carbon Feng Graphene Technology Co., Ltd., Suzhou, China. A fully automatic viscosity tester (miniAV Automatic Viscomer, Cannon, State College, PA, USA) is employed to determine the kinematic viscosity of various lubricants at room temperature. Their kinematic viscosities are listed in Table 1.

**Figure 1:**
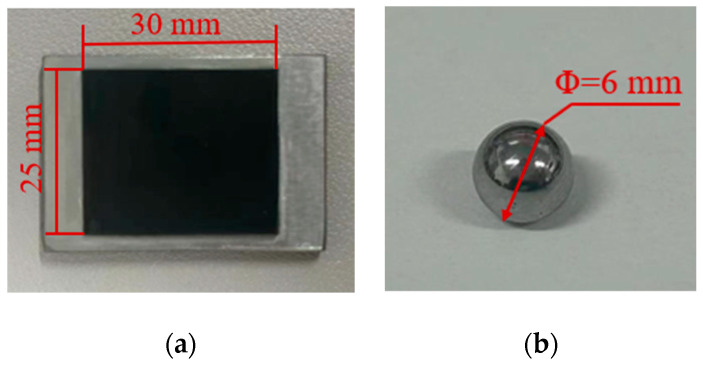
The lower and upper samples: (**a**) S45C steel sheet bonded with silicone rubber, and (**b**) 440C stainless steel ball.

The lower sample of the experiment in Figure 1a is a rectangular silicone rubber sheet with thickness of 1 mm bonded to a rectangular S45C steel sheet. The glue used for pasting is 3M quick-drying glue (AD118). After being cured for 24 h, the tensile strength of glue is about 34 MPa. The thickness of the steel sheet is 3.2 mm. The material parameters are Poisson’s ratio 0.29, Young’s modulus 200 GPa, density 7.85 g/cm^3^, and surface roughness 0.8 μm. Figure 1b shows the upper sample, which is a ball made of 440C stainless steel, with Poisson’s ratio 0.29, Young’s modulus 210 GPa, density 7.85 g/cm^3^, and surface roughness 0.06 μm. The Rockwell hardness of the 440C stainless steel ball is 58 HRC. The hardness of steel ball is much higher than that of silicone rubber.

### 2.2. Fretting Wear Experimental Procedure

**Figure 2:**
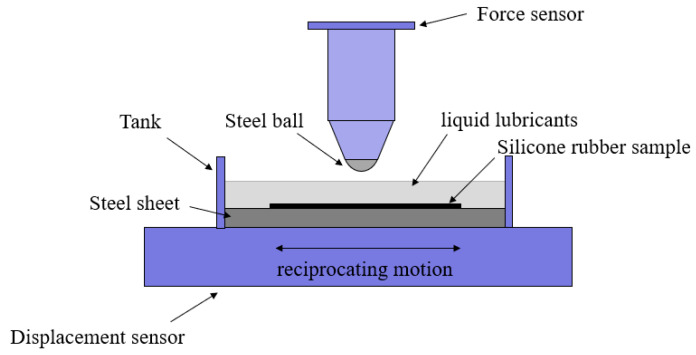
The schematic of the fretting experiment setup.

Before the experiment began, steel balls and silicone rubber samples were placed in an ultrasonic processor and washed with deionized water for 5 min. The reciprocating fretting wear experiment under sphere-on-flat contact was carried out with a reciprocating friction wear tester (Bruker UMT-Tribolab, Billerica, MA, USA). Figure 2 shows the diagrammatic sketch of the experiment setup. The force sensor and displacement sensor were employed to measure real-time normal force, tangential force, and tangential displacement. The silicone rubber pasted on the steel sheet was fixed in the tank located in the lower part of the tester. For liquid lubrication experiment conditions, a quantity of liquid lubricant was injected into the tank, so that the silicone rubber was completely immersed in the liquid lubricant. The steel ball was fixed horizontally. A specified normal force was applied to the ball to move it downward and make contact with the silicone rubber sample. Then, the normal force remained constant, and the lower sample was reciprocated in the form of a sine function.

The experiment was conducted at a room temperature of 25 °C. The parameters of wear teats are shown in Table 2 [37,38]. In order to reduce experimental errors, three repeated experiments were carried out to obtain average results.

During the experiment, the tester recorded the instantaneous tangential force *F_t_*, relative tangential displacement *D*, and normal force *F_n_* with 50 points for each cycle. At stable sample points [39], the real-time COF was calculated by using a formula *μ* = *F_t_*/*F_n_*. Then, the Savitzky–Golay smoothing method was employed to process the raw data of COF, see Savitzky and Golay [40] and Shu et al. [39] for details.

**Figure 3:**
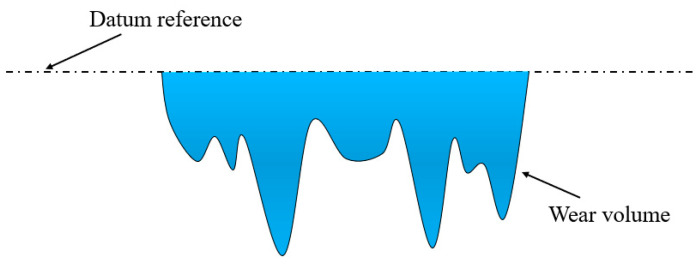
The schematic diagram for calculating the wear volume.

Scanning electron microscopy (SEM, ZEISS Sigma 300, Oberkochen, Baden-Württemberg, Germany) was employed to observe surface morphologies. The 3D surface wear profile was characterized by a 3D white-light interferometric profilometer (ZeGage Plus, Zygo Corporation, Middlefield, CT, USA). The profilometer could directly measure the wear volume. Firstly, the 3D surface profile of the contact area was obtained by measuring the height of each point on the surface. Then, the average height of all points outside the wear area was used as the reference height. Finally, the software of the profilometer was used to integrate all points below the reference height to obtain the wear volume. The schematic diagram for calculating the wear volume is plotted in Figure 3.

## 3. Results and Discussion

### 3.1. Tangential Force and Relative Tangential Displacement Curves

The tangential force and relative tangential displacement (*F_t_-D*) curve reflects the fretting behavior and motion state of the friction interface, which is the most basic information in the fretting experiment [10]. In general, we can obtain three different shapes of *F_t_-D* curves, i.e., parallelogram, straight, and elliptic. When the normal force *F_n_* is large or the displacement amplitude *d* is small, the fretting process usually runs in the partial slip region, and *F_t_-D* curves are straight or elliptic [41]. The contact area can be significantly separated into two parts: the slip area at the contact edge and the adhesion area at the contact center. When *F_t_-D* curves are parallelograms, the fretting process runs in the gross slip region, and two contact surfaces in the whole contact area experience relative sliding. When the straight, elliptic, and parallelogram *F_t_-D* curves alternate, the fretting process runs in the mixed fretting region. In addition to the properties of the material itself, there are many factors influencing the fretting process, such as the displacement amplitude and normal force. The area enclosed by *F_t_-D* curves during the fretting process represents the energy dissipation [42].

**Figure 4:**
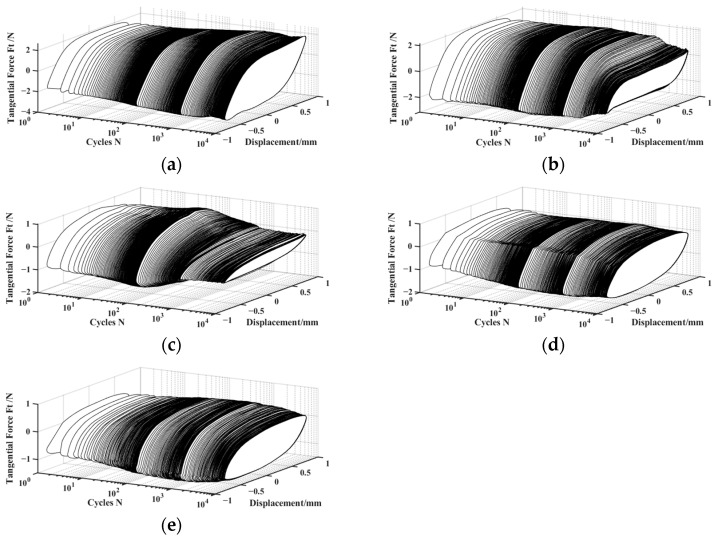
*F_t_-D-N* curves with *d* = ± 0.8 mm, *F_n_ =* 5 N, and *f* = 10 Hz: (**a**) dry friction, (**b**) water, (**c**) engine oil, (**d**) DSO, and (**e**) DSO/GO.

**Figure 5:**
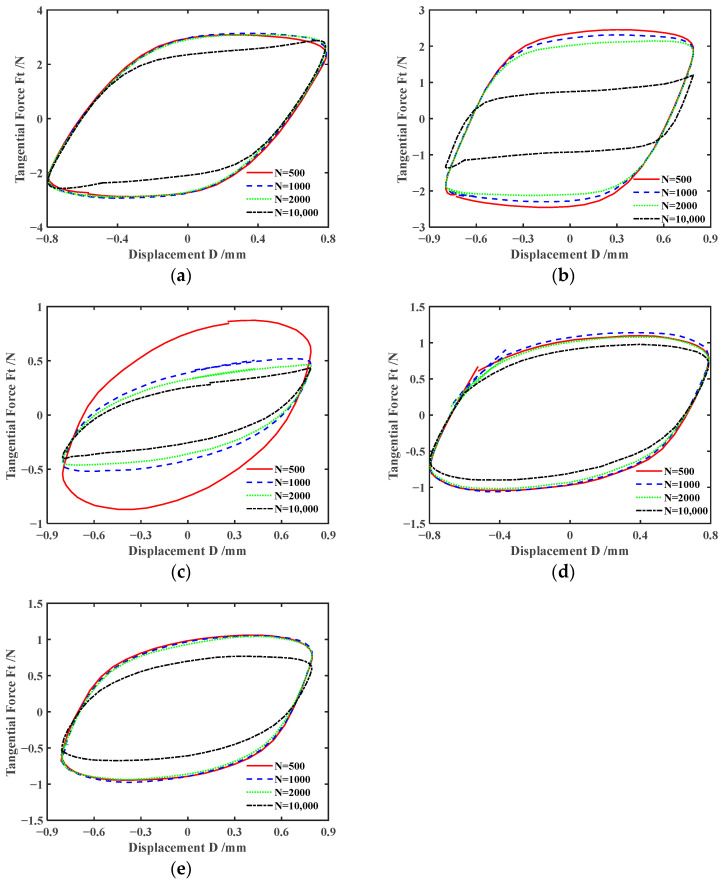
*F_t_-D* curves with *d* = ± 0.8 mm, *F_n_ =* 5 N, and *f* = 10 Hz: (**a**) dry friction, (**b**) water, (**c**) engine oil, (**d**) DSO, and (**e**) DSO/GO.

The three-dimensional *F_t_-D-N* curve describes the variation of the tangential force *F_t_* vs. relative tangential displacement *D* as a function of the number of cycles *N*. It can reflect the variation during the entire fretting process. The two-dimensional *F_t_-D* curve describes the change in tangential force *F_t_* with relative tangential displacement *D* under a fixed cycle. Figure 4 shows the *F_t_-D-N* curves of silicone rubber under dry friction and different lubrication conditions during the entire fretting process (the number of cycles *N* ranges from 1 to 10,000). Figure 5 displays the corresponding *F_t_-D* curves under four fixed cycles (*N =* 500, 1000, 2000, 10,000). Under both dry friction and various lubrication conditions, *F_t_-D* curves exhibit the parallelogram shape, and the fretting process runs in the gross slip region. It can be seen that the changes in lubrication conditions have little effect on the fretting state. Under the dry friction condition, the *F_t_-D* curves always exhibit a nearly overlapping parallelogram shape during the whole of the fretting process. And the area of the parallelogram decreases slightly only in the final stage of experiment. Under the water lubrication condition, when the fretting experiment runs around the 4000th cycle, the maximum tangential force *F_t_* suddenly decreases significantly. And the *F_t_-D* curves become a flatter parallelogram. This indicates that the energy dissipation of the fretting is reduced. Under the engine oil lubrication condition, when the fretting reaches about the 200th cycle, the *F_t_-D* curves begin to gradually contract. And the shape of *F_t_-D* curves tends to stabilize at 1000th cycle. Finally, the shape of *F_t_-D* curves gradually becomes a very flat parallelogram. Correspondingly, after the 200th cycle, the maximum tangential force starts to decrease significantly, and reaches basic stability after the 1000th cycle. Subsequently, as the fretting cycle increases, the maximum tangential force slightly decreases. The changes in *F_t_-D* curves often correspond to complex dynamic processes, involving self-cleaning and the re-infiltration of lubricants, separation and fragmentation of the wear layer, etc. [43]. The rapid decrease in tangential force may be due to more lubricants penetrating into the contact surface through fretting. When the contact area is lubricated with DSO or DSO/GO, the maximum tangential force and energy dissipation decrease slightly only in the final stage of the fretting. In addition, the tangential force *F_t_* under lubrication conditions is significantly lower than that under dry friction.

**Figure 6:**
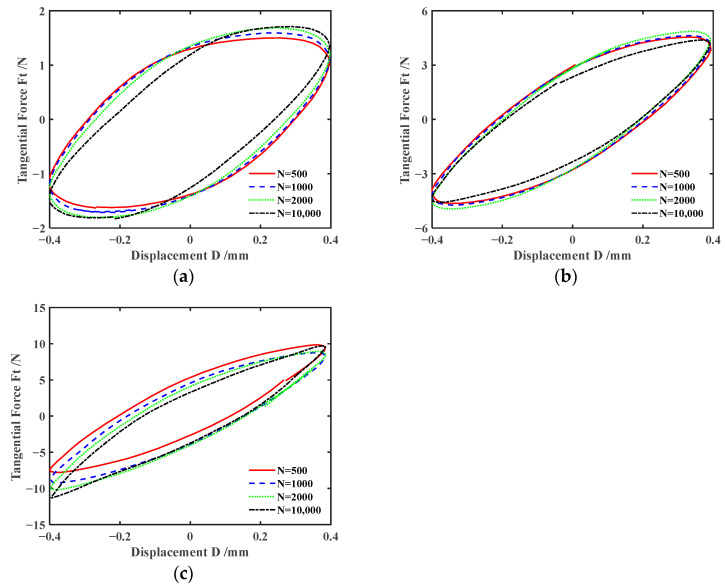
Effect of the normal force *F_n_* on *F_t_-D* curves with *d* = ± 0.4 mm and *f* = 10 Hz under engine oil lubrication condition: (**a**) *F_n_ =* 5 N, (**b**) *F_n_ =* 15 N, and (**c**) *F_n_ =* 30 N.

**Figure 7:**
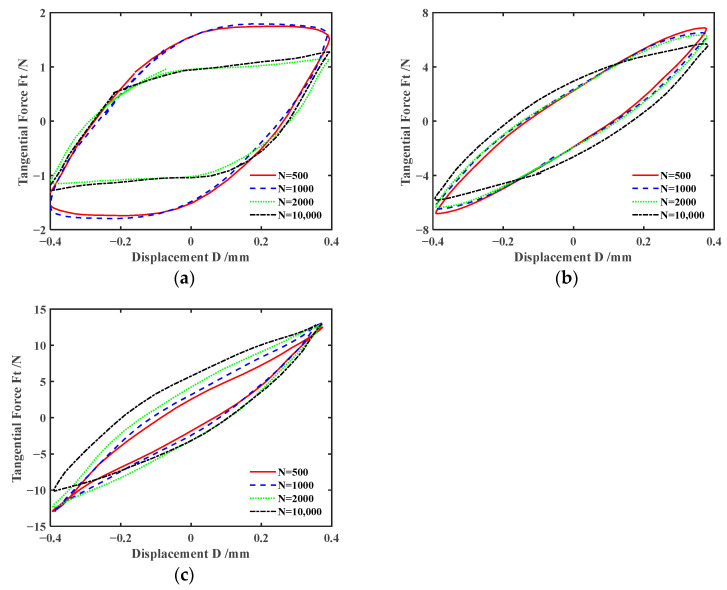
Effect of the normal force *F_n_* on *F_t_-D* curves with *d* = ±0.4 mm and *f* = 10 Hz under water lubrication condition: (**a**) *F_n_ =* 5 N, (**b**) *F_n_ =* 15 N, and (**c**) *F_n_ =* 30 N.

Figure 6 and Figure 7 plot the effect of normal force *F_n_* on *F_t_-D* curves of silicone rubber under engine oil lubrication and water lubrication conditions, respectively. The shape of *F_t_-D* curves under other lubrication and dry friction conditions is similar to that under the engine oil lubrication condition and will not be introduced here. According to Figure 6 and Figure 7, the shape of the *F_t_-D* curves is consistent under different lubrication conditions. So, the influence of the lubrication condition on the fretting state is very small. The *F_t_-D* curves always maintain a parallelogram shape for *F_n_ =* 5 N. The fretting process runs in the gross slip region. For *F_n_* = 15 N and 30 N, *F_t_-D* curves maintain an elliptical shape. The fretting process runs in the partial slip region. That is, under different lubrication conditions, the fretting transitions from the gross slip region to the partial slip region as *F_n_* increases. This is because the increase in normal force results in the increase in deformation of the silicone rubber, contact area, and tangential force. It makes sliding between the steel ball and silicone rubber more difficult. In addition, when *F_n_ =* 5 N, unlike the engine oil lubrication condition, the maximum tangential force significantly decreases between the 1000th cycle and 2000th cycle under water lubrication. The possible reason is that a large amount of water seeps into the contact region under the sliding state.

**Figure 8:**
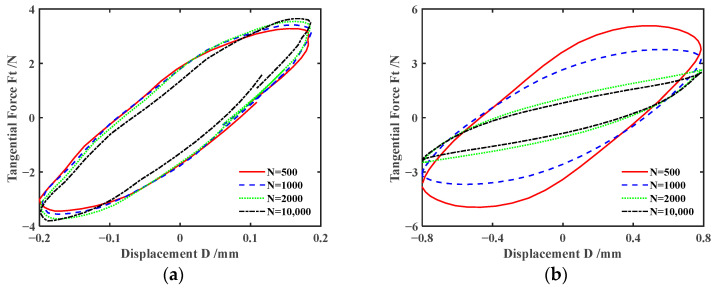
Effect of the displacement amplitude *d* on *F_t_-D* curves with *F_n_* = 15 N and *f* = 10 Hz under engine oil lubrication condition: (**a**) *d* = ±0.2 mm and (**b**) *d* = ±0.8 mm.

**Figure 9:**
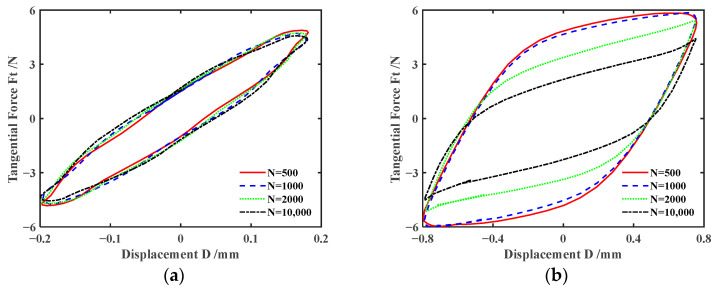
Effect of the displacement amplitude *d* on *F_t_-D* curves with *F_n_* = 15 N and *f* = 10 Hz under water lubrication condition: (**a**) *d* = ±0.2 mm and (**b**) *d* = ±0.8 mm.

Figure 8 and Figure 9 show the impact of displacement amplitude *d* on *F_t_-D* curves of silicone rubber under engine oil lubrication and water lubrication conditions, respectively. The results for *d* = ±0.4 mm are displayed in Figure 6b and Figure 7b. With the increase in the displacement amplitude, the fretting process transitions from the partial slip region to the gross slip region. This is because with the increase in the displacement amplitude, the deformation of the silicone rubber itself cannot compensate for the relative sliding between the friction pairs. Therefore, when the displacement amplitude increases, the complete slip is more likely to occur. When the displacement amplitude is large (*d* = ±0.8 mm), the energy dissipation and maximum tangential force gradually decrease as the number of cycles increases under engine oil lubrication and water lubrication conditions.

### 3.2. Coefficient of Friction

**Figure 10:**
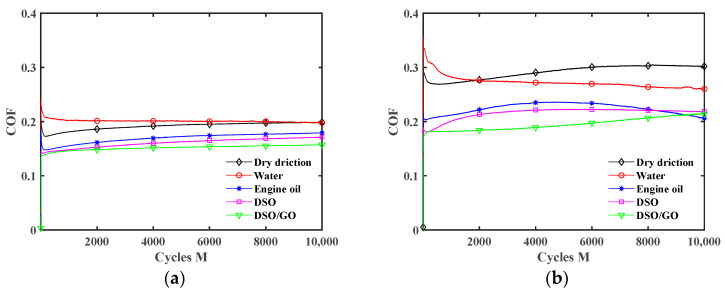

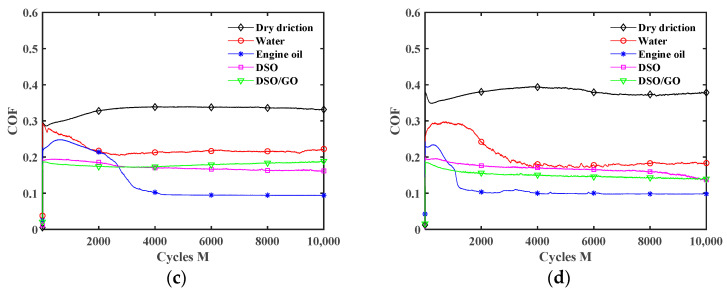
Effect of lubrication condition on the COF with *F_n_* = 15 N and *f* = 10 Hz: (**a**) *d* = ±0.2 mm, (**b**) *d* = ±0.4 mm, (**c**) *d* = ±0.6 mm, and (**d**) *d* = ±0.8 mm.

Figure 10 shows the influence of the lubrication condition on the COF under different displacement amplitudes. Throughout the whole fretting wear process, the COF curves consist of two stages. In the running-in period, the COF rapidly increases or decreases, mainly caused by damage to the smooth contact surface in the early stages of fretting. In the stable wear stage, the COF remains relatively stable but fluctuates slightly near the stable value. When the displacement amplitude is small (*d* = ±0.2 mm), the influence of the lubrication condition on the duration of the running-in period is very small. For a large displacement amplitude (*d* = ±0.6 mm and ±0.8 mm), the COF curves have a significantly longer running-in period under the water lubrication and engine oil lubrication conditions. It is because when the displacement amplitude is large, the fretting process tends to enter the gross slip state, and the lubricant on the contact surface will form a complete lubricating film. The running-in period corresponds to the formation process of a lubricating film, and wear is relatively severe during this period [44]. Compared to DSO and DSO/GO lubricants, water and engine oil lubricants have lower kinematic viscosity (see Table 1). So, it takes a longer time to form a complete lubricating film. Xie et al. [45] also found a similar phenomenon when they analyzed the effect of water content in an ionic liquid/water mixture on the running-in process of the COF curves. As the water content in the ionic lubricant increases, the running-in period of the COF curves becomes longer. Under pure water lubrication, the COF curve has the longest running-in period.

Compared to a large displacement amplitude, the COF curves in Figure 10a are very smooth under a small displacement amplitude. And the lubrication conditions have little effect on the COF curves under a small displacement amplitude. This is because the fretting process runs in the partial slip region under a small displacement amplitude (see Figure 8a and Figure 9a). In the early stage of fretting, the liquid lubricant will be squeezed out of the contact area (i.e., self-cleaning of lubricant) [43]. The steel ball and silicone rubber directly contact during the subsequent wear process. Due to the small relative sliding only at the contact edge, liquid lubricant cannot enter the contact center. And only a small amount of liquid lubricant exists at the contact edge. Therefore, under the partial slip state, the liquid lubricant only plays a small role.

For *d* = ±0.4 mm in Figure 10b, all liquid lubricants begin to take effect in reducing the COF. The effect of lubricant on the COF, from strong to weak, is in the order of DSO/GO, DSO, engine oil, and water. Due to the high kinematic viscosity of DSO and DSO/GO (see Table 1), it is easy to form a high-strength lubricating film, and the lubricating film is not easy to rupture, resulting in a smaller COF. In addition, due to the sheet-like structure of GO, it is easy for GO to enter contact area and form a lubricating film. Due to the good dispersibility of GO in liquids, it can be continuously supplied to the contact surfaces. So, when DSO/GO is used as the lubricant, the COF is the smallest.

In Figure 10c,d, the lubrication effect is more significant under a large displacement amplitude (*d* = ±0.6 mm and ±0.8 mm). For the large displacement amplitude, the fretting process runs in the gross slip region (see Figure 8b and Figure 9b). The lubricant can easily enter contact region and form a complete and thick lubricating film. The COF is the smallest under engine oil lubrication, followed by DSO and DSO/GO. This may be because the kinematic viscosity of DSO is much higher than that of engine oil. The excessive kinematic viscosity means high shear strength, leading to an increase in friction resistance. Water has the worst friction reduction effect among all lubricants, but the COF is still much smaller than that under dry friction.

**Figure 11:**
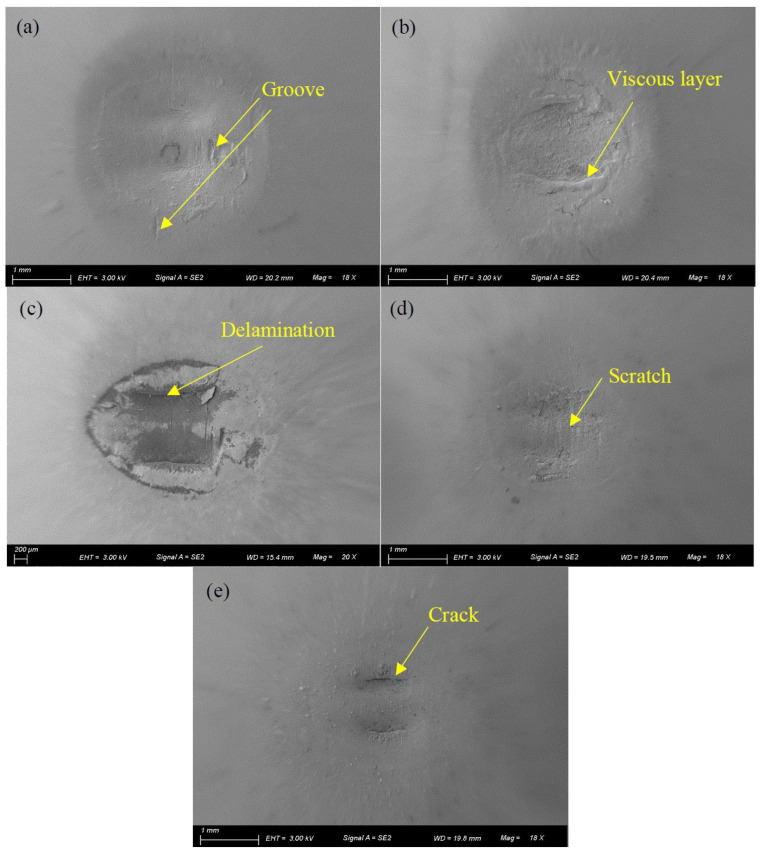
The SEM morphologies of silicone rubber with *d* = ± 0.4 mm, *F_n_* = 15 N, and *f* = 10 Hz: (**a**) dry friction, (**b**) water, (**c**) engine oil, (**d**) DSO, and (**e**) DSO/GO.

To further explain Figure 10b, Figure 11 shows the SEM morphologies of silicone rubber under different lubrication conditions with *d* = ±0.4 mm. Under dry friction, engine oil lubrication, and water lubrication conditions, the wear surface exhibits relatively severe damage. Under dry friction, there are numerous grooves on the surface. Under water lubrication, the local viscous layers are formed under the alternating stress, and the surface at the contact center is rough. Under engine oil lubrication, there is a significant delamination phenomenon on the wear surface. Under the DSO and DSO/GO lubrication conditions, the surface of wear is very smooth. There are slight scratches at the contact center under DSO lubrication and cracks on the contact surface under the DSO/GO lubrication. Thus, the COF is small under DSO and DSO/GO lubrication conditions.

**Figure 12:**
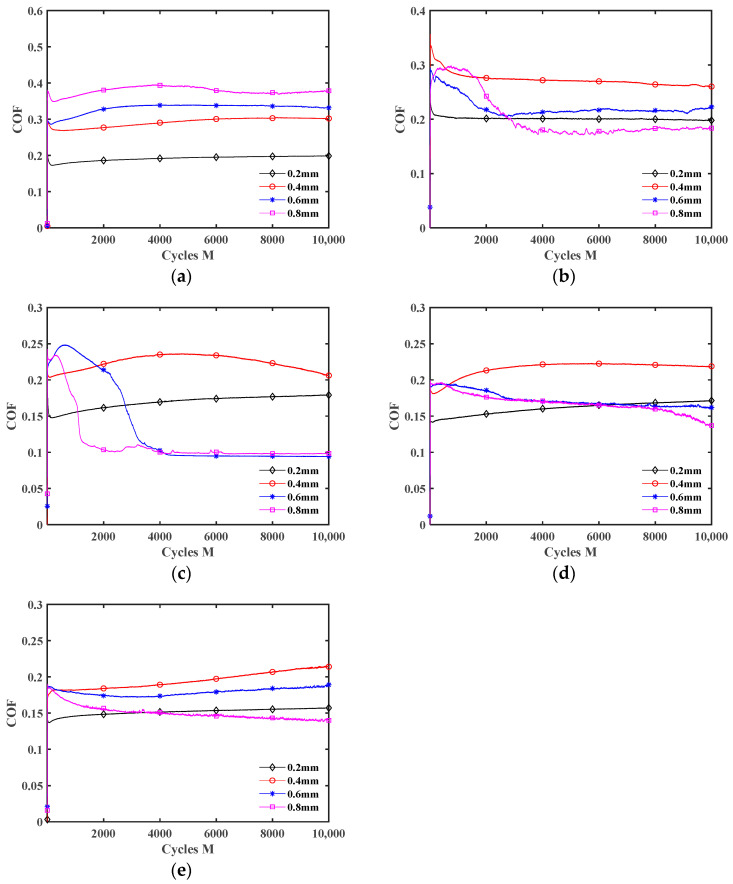
Effect of the displacement amplitude *d* on the COF with *F_n_* = 15 N and *f* = 10 Hz: (**a**) dry friction, (**b**) water, (**c**) engine oil, (**d**) DSO, and (**e**) DSO/GO.

Figure 12 plots the influence of the displacement amplitude *d* on the COF under different lubrication conditions. The fretting transitions from the partial slip state to the gross slip state with the increment of displacement amplitude, so the COF increases under dry friction. The COF increases first and then decreases as *d* increases under liquid lubrication conditions. It is because when the displacement amplitude is small (*d* = ±0.2 mm and ±0.4 mm), the fretting is in the partial slip state (see Figure 6b, Figure 7b, Figure 8a and Figure 9a), so the lubrication effect of the liquid lubricants is very small. Thus, the influence of *d* on the COF is the same as under dry friction. When *d* = ±0.6 mm and ±0.8 mm, the lubricants form an effective lubricating film, so the COF significantly decreases. In addition, the COF curves under the dry friction condition are very smooth during the stable wear stage. However, when *d* = ±0.6 mm and ±0.8 mm, there is a significant oscillation in the COF curves under the water lubrication condition. The reason is that owing to the low kinematic viscosity of water, the strength of the lubricating film is low. So, the lubricating film is more prone to failure during wear [46]. In other words, during the fretting process, a competition between the self-cleaning and re-penetration of lubricants occurs. When more lubricant penetrates into the contact interface, it will result in a rapid reduction in the COF. Due to the strong self-cleaning of the lubricant at the contact interface, it can lead to a rapid increase in the COF. Due to this competition, the COF curves will experience significant fluctuations. However, under the engine oil lubrication, DSO lubrication, and DSO/GO lubrication conditions, the COF curves did not show significant oscillation. This is because the high kinematic viscosity of engine oil and DSO makes the lubricating film easy to form and not easy to be damaged.

**Figure 13:**
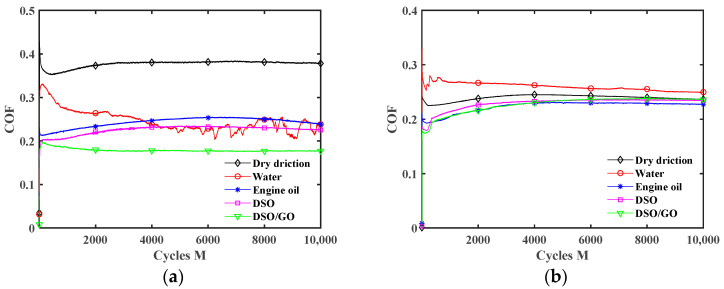
Effect of lubrication condition on the COF with *d* = 0.4 mm and *f* = 10 Hz: (**a**) *F_n_ =* 5 N and (**b**) *F_n_ =* 30 N.

Figure 13 plots the effect of the lubrication condition on the COF under different normal forces. The results for *F_n_ =* 15 N are given in Figure 10b. As *F_n_* increases from 5 N to 30 N, the fretting changes from the gross slip state to the partial slip state (see Figure 6 and Figure 7). So, the difference in the COF is relatively small under different lubrication conditions for *F_n_ =* 30 N. When *F_n_* is small (5 N and 15 N), the lubrication effect of the liquid lubricant is significant. The results for *F_n_ =* 5 N and 15 N are similar. However, unlike *F_n_ =* 15 N, since the fretting state has more slip when *F_n_ =* 5 N, the COF curve exhibits significant oscillation under water lubrication. The cause of oscillation is owing to the fact that water has the lowest kinematic viscosity among these liquid lubricants, leading to the continuous self-cleaning and re-penetration of water at the contact interface during the fretting.

**Figure 14:**
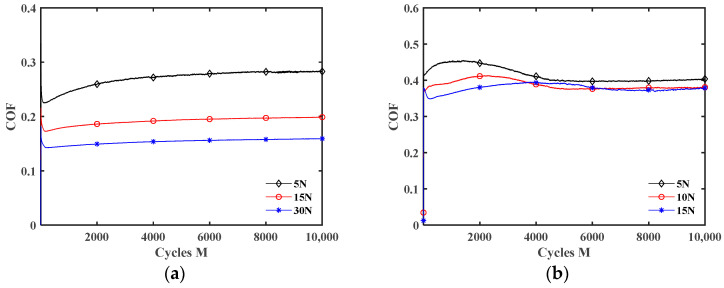
Effect of the normal force *F_n_* on the COF under dry friction condition with *f* = 10 Hz: (**a**) *d* = ± 0.2 mm and (**b**) *d* = ± 0.8 mm.

**Figure 15:**
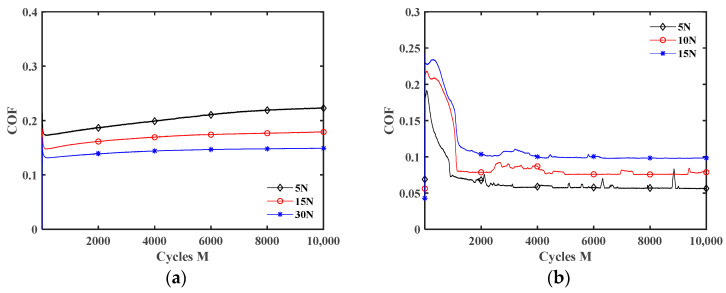
Effect of the normal force *F_n_* on the COF under engine oil lubrication condition with *f* = 10 Hz: (**a**) *d* = ±0.2 mm and (**b**) *d* = ±0.8 mm.

**Figure 16:**
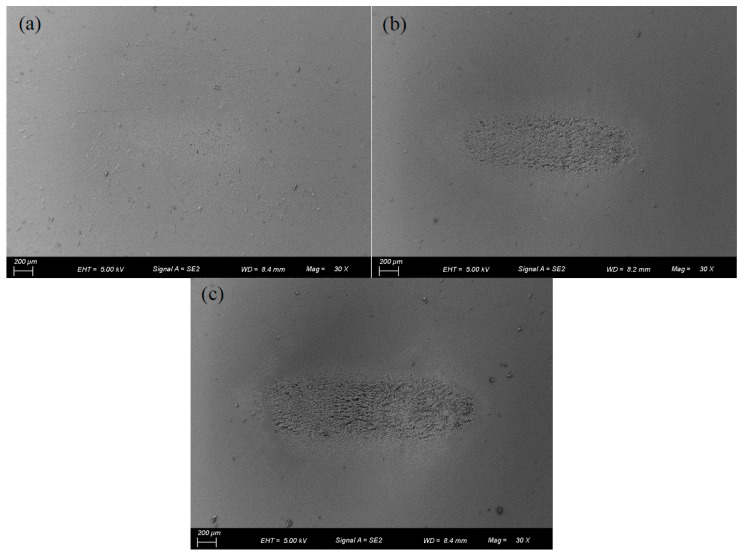
The SEM morphologies of silicone rubber under engine oil lubrication with *d* = ±0.8 mm and *f* = 10 Hz: (**a**) *F_n_* = 5 N, (**b**) *F_n_* = 10 N, and (**c**) *F_n_* = 15 N.

Figure 14 and Figure 15 show the effect of *F_n_* on the COF under dry friction and engine oil lubrication conditions, respectively. The influences of *F_n_* on the COF under water, DSO, and DSO/GO lubrication conditions are very similar to that under the engine oil lubrication condition. When *d* = ±0.2 mm, the COF decreases with the increase in *F_n_* under both the dry friction and liquid lubrication conditions. The reason may be that it is difficult for lubricants to enter the contact area under a small displacement amplitude, and the lubrication effect is very limited. Therefore, the influences of *F_n_* on the COF are the same for dry friction and liquid lubrication. In addition, as the normal force increases, the fretting state transitions from gross slip to partial slip (see Figure 6 and Figure 7), resulting in a decrease in the COF. In addition, when *d* = ±0.8 mm, the influence of *F_n_* on the COF is very small under the dry friction condition. However, under the engine oil lubrication condition, the COF increases with the increase in *F_n_*. When *d* = ±0.8 mm, the fretting is in the gross slip state (see Figure 5, Figure 8b and Figure 9b). The liquid lubricant can fully enter contact surface and form a lubrication film under the gross slip state. So compared to dry friction condition, the COF is significantly reduced under the engine oil lubrication condition. When fixed *d* = ±0.8 mm, increasing the normal force will cause the fretting to become more adhesive and the lubricating film to become thinner. This prevents the lubrication film from effectively protecting the contact surface, resulting in an increase in the COF in Figure 15b. This can also be seen from the SEM morphologies. Figure 16 shows the SEM morphologies of silicone rubber with different normal force under engine oil lubrication condition when *d* = ±0.8 mm. When *F_n_ =* 5 N, the contact surface is very smooth. However, as the normal force increases, the wear surface of silicone rubber becomes rougher. And the area and depth of the wear area increase.

### 3.3. Surface Morphology and Wear Mechanism

**Figure 17:**
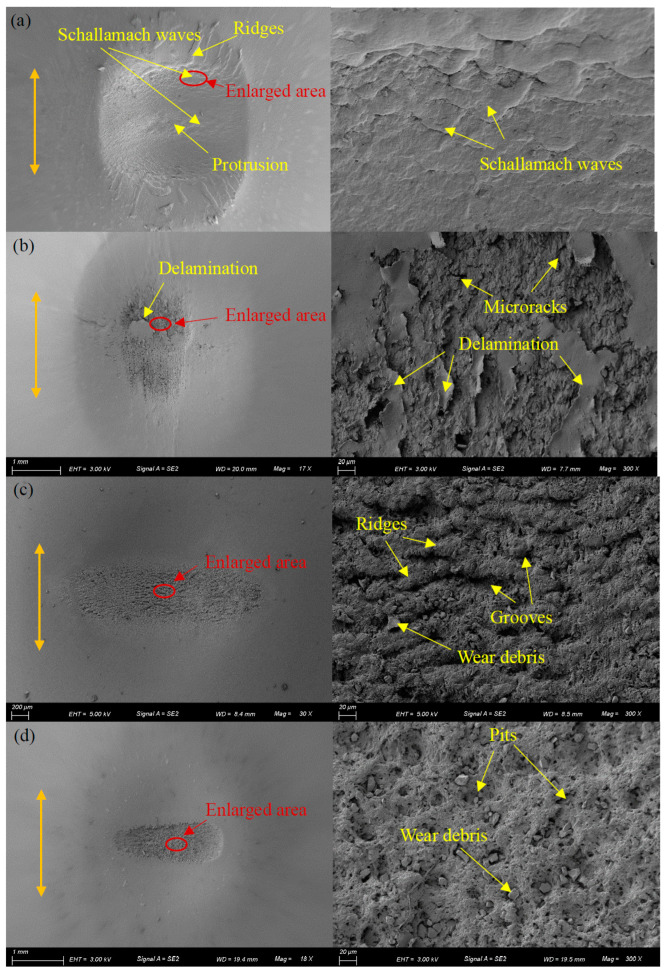

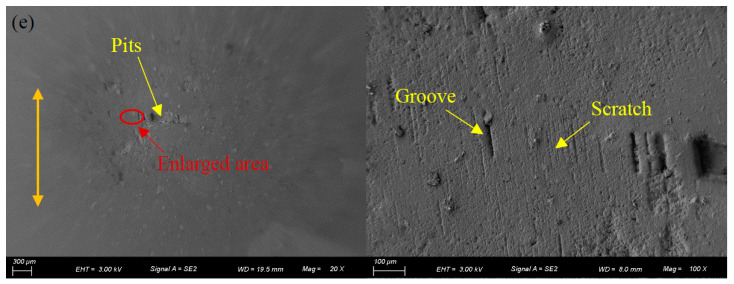
The SEM morphologies of silicone rubber with *d* = ±0.8 mm, *F_n_* = 15 N, *f* = 10 Hz, and *N* = 10^4^: (**a**) dry friction, (**b**) water, (**c**) engine oil, (**d**) DSO, and (**e**) DSO/GO.

Figure 17 demonstrates SEM morphologies of the surface features of silicone rubber under different lubrication conditions. The panel on the left displays the overall view of the wear region, while panel on the right displays a partially enlarged view within the red mark. Under dry friction, there are numerous waves perpendicular to the fretting direction on the wear center, known as Schallamach pattern wear [47]. These waves are caused by adhesion force. There are obvious elongated rubber ridges at the contact edge, which are not separated from the rubber base. At the contact center, there is a slight protrusion. Under dry friction, adhesive wear is the main form of wear. Under water lubrication, the rubber at the contact center is peeled off in large areas, resulting in an obvious delamination phenomenon on the wear surface. After the rubber surface is peeled off, many microcracks appear on the wear surface. Due to the strong permeability of water, it is easy for water to penetrate into the already formed microcracks. It will promote crack growth and cause severe wear. Adhesive wear and fatigue wear are the main forms of fretting wear under water lubrication. Under engine oil lubrication, there are numerous ridges and grooves at the contact center, which are perpendicular to the direction of fretting. There are many small examples of wear debris on the surface. However, at both contact ends, the surface is very smooth. The wear form is principally abrasive wear, fatigue wear, and adhesive wear under engine oil lubrication. Similar to engine oil lubrication, there is significant wear at the contact center under DSO lubrication. Many dotted pits and examples of wear debris are found at the contact center. The wear form is similar to engine oil lubrication. The surface damage is lighter under the DSO/GO lubrication condition, with only slight scratches, grooves, and pits. This is attributed to the protective effect played by GO. There is only abrasive wear on the wear surface under DSO/GO lubrication.

### 3.4. Wear Volume

**Figure 18:**
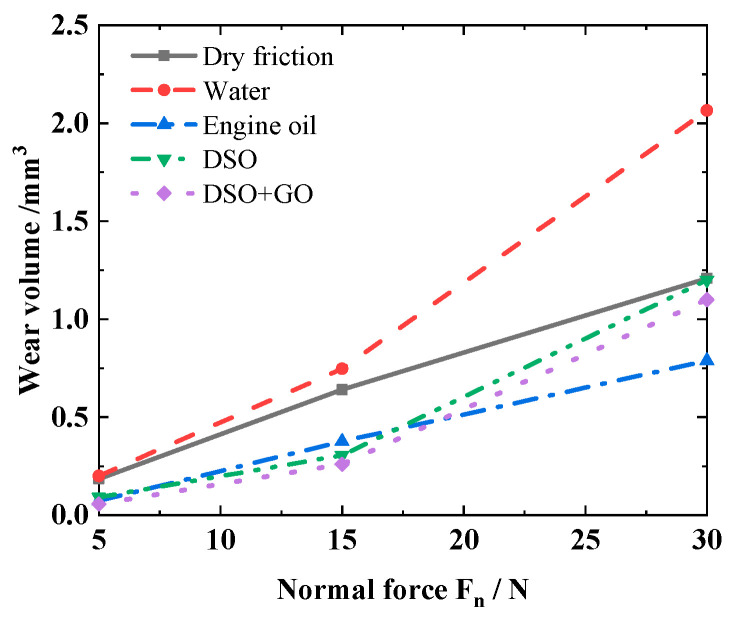
Effect of the normal force *F_n_* on the wear volume with *f* = 10 Hz, *d* = ± 0.4 mm, and *N =* 10^4^.

Figure 18 presents the variation in the wear volume of silicone rubber with normal force *F_n_* under different lubrication conditions. It is seen that as the *F_n_* increases, the wear volume always increases. Compared to dry friction, the wear volume is greater under water lubrication. The reason might be that during the fretting wear process, defects and microcracks appear on the contact surface (see Figure 17b). Water can easily enter these defects and microcracks, causing crack propagation and more severe wear [23]. Similar results can be found from Yu et al. [48] and Srinath and Gnanamoorthy [49]. In comparison with dry friction, the wear volume of silicone rubber decreases to varying degrees under other lubrication conditions. For a small normal force, the wear volume of silicone rubber is the smallest under DSO/GO lubrication, followed by DSO lubrication and engine oil lubrication. The reason may be that the fretting process runs in the gross slip region under a small normal force. A complete lubricating film can be formed on the contact surface. DSO has a higher kinematic viscosity, resulting in a higher strength of the lubrication film and stronger protective effect on the silicone rubber. Moreover, due to the advantages of the high strength and smooth surface of GO, its addition to lubricants can provide good protection. So, the wear volume of silicone rubber is smaller under the DSO/GO lubrication condition than that under the DSO lubrication condition. However, for a large normal force, the wear volume under engine oil lubrication is smaller. When the normal force is high, the fretting process runs in the partial slip region. At this time, it is easier for the engine oil to enter the contact surface by virtue of the lower kinematic viscosity, providing a certain degree of protection.

**Figure 19:**
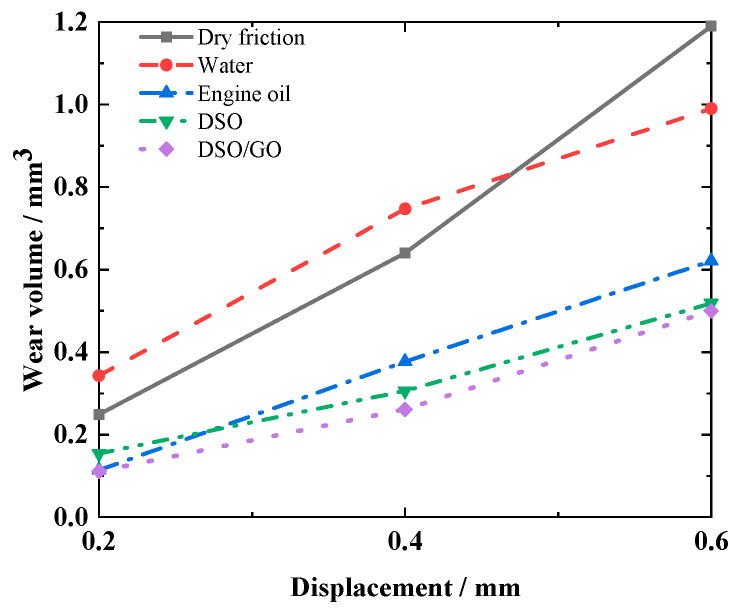
Effect of the displacement amplitude *d* on the wear volume with *f* = 10 Hz, *F_n_* = 15 N, and *N =* 10^4^.

Figure 19 shows the impact of the *d* on wear volume of silicone rubber under different lubrication conditions. Under various lubrication and dry friction conditions, as the *d* increases, the wear volume increases. The reason is that as *d* increases, fretting is more likely to enter a slip state, and the area of wear increases. In addition to the water lubrication condition, wear volume can be significantly reduced under other liquid lubrication conditions. Under the DSO/GO lubrication condition, the wear volume is the smallest.

## 4. Conclusions

The fretting wear behaviors of silicone rubber are studied experimentally under dry friction and different lubrication conditions. Four lubrication conditions are considered, including water, engine oil, DSO, and DSO/GO. The influences of normal force, lubrication condition, and displacement amplitude on friction curves, COF, damage characteristic, wear volume, and fretting wear performance of silicone rubber are discussed. The following can be found:

(1) As the displacement amplitude increases or the normal force decreases, the fretting process transitions from the partial slip region to the gross slip region. The change in lubrication conditions has little effect on the fretting state.
(2) Liquid lubrication can significantly reduce the COF, especially when the normal force is small or displacement amplitude is large. For a small displacement amplitude, the COF of silicone rubber is the smallest under DSO/GO lubrication. For a large displacement amplitude, the COF is the smallest under engine oil lubrication.
(3) As the displacement amplitude increases, COF increases under the dry friction condition, but it first increases and then decreases under liquid lubrication conditions.
(4) Under the dry friction condition, the COF decreases as the normal force increases. Under liquid lubrication conditions, with the increase in the normal force, the COF decreases for a small displacement amplitude but increases for a large displacement amplitude.
(5) Water has the worst friction reduction effect among all lubricants, but the COF is still much smaller than that under dry friction. The wear volume is greater for water lubrication compared with dry friction.
(6) When the normal force is small, the wear volume of silicone rubber is the smallest under DSO/GO lubrication, followed by DSO lubrication and engine oil lubrication. When the normal force is large, the wear volume is the smallest under engine oil lubrication, followed by DSO/GO lubrication and DSO lubrication.

The present paper makes the first attempt to experimentally study the fretting wear behaviors of silicone rubber under dry friction and different liquid lubrication conditions. The limitations of this study include two aspects: (1) The influence of temperature is not taken into account, and in fact, rubber seals are often used in high-temperature environments. (2) No numerical simulation is conducted because the introduction of fluid–solid coupling will bring great difficulties to the simulation. In the future, it can be extended to account for the influences of (1) temperature, (2) silicone rubber matrix composites adding particles or fibers, and (3) biomimetic surface texture.

## Figures and Tables

**Table 1:** The kinematic viscosity of the liquid lubricants.

Lubricants	Kinematic Viscosity (mm^2^/s) at 25 °C
Water	0.893
Engine oil	130.6
DSO	1049.6
DSO/GO	1089.2

**Table 2:** The parameters of the wear teats.

Parameter	Range
Frequency	*f* = 10 Hz
Displacement amplitude	*d* = ±0.2 mm, ±0.4 mm, ±0.6 mm, ±0.8 mm
Normal force	*F_n_* = 5 N, 10 N, 15 N, 30 N
Number of cycles	*N* = 10^4^

## Data Availability

All data, models, or code generated or used during the study are available from the corresponding author by request.
